# Results of a psychosomatic training program in China, Vietnam and Laos: successful cross-cultural transfer of a postgraduate training program for medical doctors

**DOI:** 10.1186/1751-0759-6-17

**Published:** 2012-08-29

**Authors:** Kurt Fritzsche, Peter Scheib, Nayeong Ko, Michael Wirsching, Andrea Kuhnert, Jie Hick, Gerhard Schüßler, Wenyuan Wu, Shen Yuan, Nguyen Huu Cat, Sisouk Vongphrachanh, Ngo Tich Linh, Ngyuen Kim Viet

**Affiliations:** 1 Department of Psychosomatic Medicine and Psychotherapy, University Medical Center, Freiburg, Germany; 2 Department of Medical Psychology and Psychotherapy, University Hospital, Innsbruck, Austria; 3 Tongji Hospital of Tongji University, Shanghai, China; 4 University Medical College, Hue, Vietnam; 5 Mental Health Department, National University of Laos, Vientianne, Laos; 6 University of Medicine and Pharmacy, Ho Chi Minh City, Vietnam; 7 Medical University, Hanoi, Vietnam

**Keywords:** Psychosomatic medicine, Curriculum, Teaching of teachers, China, Vietnam, Laos

## Abstract

**Background:**

With the “ASIA-LINK” program, the European Community has supported the development and implementation of a curriculum of postgraduate psychosomatic training for medical doctors in China, Vietnam and Laos. Currently, these three countries are undergoing great social, economic and cultural changes. The associated psychosocial stress has led to increases in psychological and psychosomatic problems, as well as disorders for which no adequate medical or psychological care is available, even in cities. Health care in these three countries is characterized by the coexistence of Western medicine and traditional medicine. Psychological and psychosomatic disorders and problems are insufficiently recognized and treated, and there is a need for biopsychosocially orientated medical care. Little is known about the transferability of Western-oriented psychosomatic training programs in the Southeast Asian cultural context.

**Methods:**

The curriculum was developed and implemented in three steps: 1) an experimental phase to build a future teacher group; 2) a joint training program for future teachers and German teachers; and 3) training by Asian trainers that was supervised by German teachers. The didactic elements included live patient interviews, lectures, communication skills training and Balint groups. The training was evaluated using questionnaires for the participants and interviews of the German teachers and the future teachers.

**Results:**

Regional training centers were formed in China (Shanghai), Vietnam (Ho Chi Minh City and Hue) and Laos (Vientiane). A total of 200 physicians completed the training, and 30 physicians acquired the status of future teacher. The acceptance of the training was high, and feelings of competence increased during the courses. The interactive training methods were greatly appreciated, with the skills training and self-experience ranked as the most important topics. Adaptations to the cultural background of the participants were necessary for the topics of “breaking bad news,” the handling of negative emotions, discontinuities in participation, the hierarchical doctor-patient relationship, culture-specific syndromes and language barriers. In addition to practical skills for daily clinical practice, the participants wanted to learn more about didactic teaching methods. Half a year after the completion of the training program, the participants stated that the program had a great impact on their daily medical practice.

**Conclusions:**

The training in psychosomatic medicine for postgraduate medical doctors resulted in a positive response and is an important step in addressing the barriers in providing psychosomatic primary care. The transferability of western concepts should be tested locally, and adaptations should be undertaken where necessary. The revised curriculum forms the basis of training in psychosomatic medicine and psychotherapy for medical students and postgraduate doctors in China, Vietnam and Laos.

## Background

In different ways, China, Vietnam and Laos have undergone major social, economic and cultural changes. Traditional values are being questioned or are disappearing, and new social values and structures have not yet been established. Despite significant increases in the wealth of the population, particularly in the cities, there have been simultaneous increases in uncertainty and in stressful living situations for a large majority of the population. As a result of these social upheavals, psychological and psychosomatic disorders and problems are on the rise [1-5].

In Southeast Asia, 11% of disability-adjusted life years and 27% of years lived with disabilities are the results of neuropsychiatric disease [3]. Depression is the largest contributor to this disease burden [1]. The majority of the patients who suffer from common mental disorders (CMDs), such as depressive and anxiety disorders, seek help in primary care [1]. The point prevalence of CMDs in South Asian primary care practices varies between 20 and 45%. A review of 8 epidemiological studies of common mental disorders in South Asia showed that their point prevalence in primary care was 26.3% (95% CI, 25.3%-27.4%). Less than one third of clinically significant CMDs are recognized [6-9]. The resulting chronicity causes severe burdens on the affected families and on the health system.

In contrast, East Asia, particularly in China, has a health system that is based on high-tech Western medicine. Due to missing or insufficiently developed health insurance systems, only patients with sufficient incomes can afford this extremely expensive care. To a limited extent, the poorer classes also make use of Western medicine; however, to a large extent, they also resort to traditional medicine. Basic care is lacking for common mental and psychosomatic disorders and problems, and this care is not adequately addressed by traditional medicine. In the current medical education systems in China and Vietnam, psychosomatic or psychological content is only marginally taught [10-14]. More psychiatric education is available; however, it is taught mostly, if not always, in a biological manner, and there is a strong emphasis on psychotropic drugs and custodial care. Psychiatric resources are also lacking, which often allows the treatment of only severe cases, such as psychosis. In Vietnam, there is one psychiatrist for every 300,000 inhabitants. Only half of the eight medical schools have first-degree specialty training programs in psychiatry. Psychologists administer psychological tests and sometimes perform consultations, primarily in schools and industry [11,12,14]. Furthermore, education about consultation-liaison psychiatry and psychosomatics in daily clinical practice is nonexistent [9,15]. The situation in Laos is even worse: for the entire country, there are only two trained psychiatrists in the capital, Vientiane.

It is apparent that the current medical education systems in China, Vietnam and Laos need support in different ways to develop efficient educational institutions and care structures.

From 2005 to 2008, a European-Asian cooperative project funded by the European Union was coordinated by Freiburg University (Department of Psychosomatic Medicine and Psychotherapy). The objective of the project was to support the development of psychosomatic medicine in China (Tongji University), Vietnam (Universities of Ho Chi Minh City and Hue) and Laos (University of Vientiane). In this project, a basic psychosomatic care curriculum for postgraduate medical doctors was applied. In this report, we present the results of an evaluation of the project after 3 years of implementation and describe the necessary adaptations to the respective cultures of each of these 3 countries.

### Teaching and learning objectives

The theoretical basis of the curriculum is the biopsychosocial model system [16]. This system describes the interactions among the biological, psychological and social processes that are involved, to different extents, in each disease. The content and didactic implementation of the curriculum was guided by trainers from Germany, where basic knowledge about recognizing psychological and psychosomatic disorders and problems, counseling and providing emotional support and providing referrals to health specialists are included in the training of medical students and postgraduate doctors. The objectives of the psychosomatic approach are to build bridges between the various clinical disciplines to overcome the mind-body dichotomy and to stress the importance of understanding the interactions among biology, psychology, and social factors in every patient, independent of the primary pathology that is being treated. These objectives imply both a system-based perspective and knowledge of the biological, psychological, and social subsystems and their interactions. The psychosomatic approach focuses on the doctor-patient relationship and on an integrative strategy for diagnosing and treating patients. Educating and training the somatic clinician to integrate psychosomatic aspects of medical care into his/her daily work has become a well-accepted priority for training and research.

Germany offers three levels of psychosocial medical services. The first is basic psychosomatic care, in which medical specialists (from general medicine to urology) receive training and for which they can be reimbursed by health insurance providers. The next level consists of more advanced training in psychosomatic and psychotherapeutic skills, which is open to all disciplines (e.g., general medicine, internal medicine, gynecology and dermatology). This level requires an additional two years of training beyond the usual residency training requirements. Finally, there is a third level, specialist training in psychiatry and psychosomatic medicine, both of which require five years of instruction.

### Basic psychosomatic care

Basic psychosomatic care is rooted in psychosomatic medicine and has primarily been influenced by psychoanalysts and internists who emulated Balint’s approach as proposed in 1964 (Balint 1964), which stressed the integration of psychosomatic and holistic perspectives in the medical practice model. Basic psychosomatic care is not psychotherapy in primary care; rather, it is integrated, biopsychosocial treatment that includes the following advantages

1. The physical examination is integrated into the consulting hour. Beginning with the patient's presentation of complaints, the doctor assesses both somatic and emotional concerns. As a result, both physical and psychosocial problems are addressed in diagnosis and treatment.

2. Many patients do not feel as embarrassed about conversations regarding mental or interpersonal conflicts in the primary care setting as they might if referred to a mental health professional.

3. Conversations in the primary care setting usually occur in the context of long-standing, trusting doctor-patient relationships. Such relationships have been shown to be important factors in the healing process. Family conflicts and past crises are usually familiar to the doctor who treats the entire family. When a new conflict or symptom arises, it can be placed in a personalized context.

### Objectives of psychosomatic basic care

The four targeted skills of psychosomatic basic care include the following:

1. Identifying stressful emotional and mental disorders and conflicts using a psychosocial anamnesis;

2. Promoting a helping alliance among the doctor, patient, and family members; this skill also includes identifying possible barriers on the part of the doctor, patient, or family and stressing the core skills of empathy and sensitivity;

3. Improving the patient’s problem-solving skills, including providing information about self-help groups, supporting the management of adverse life events (e.g., severe illness, loss, separation or divorce) and avoiding unnecessary medication, diagnostic procedures, and surgery; and

4. Motivating/referring patients for psychotherapy. Additional skills in this area include collaborations around consultations and case management with psychotherapists and other psychosocial service providers.

### Targeted skill 1: Disease patterns

The selection of mental disease to be taught depends on the frequencies of the diseases’ occurrences in Southeast Asian countries [1,6-9]. The following diseases have been deemed important: 1. the various forms of depression; 2. somatoform disorders; and 3. psychological reactions, such as anxiety and adaptation disorders, to severe life-threatening diseases, such as cancer. Additional modules are available with regard to anxiety disorders, post-traumatic stress disorder, addiction and dependence. Psychopharmacological treatment approaches have been integrated into the education about each disease pattern.

### Targeted skills 2 and 3: Interventions

The basic therapeutic approaches integrate psychodynamic approaches, cognitive-behavioral models, systemic family therapy and communication skills, such as empathy, unconditional positive regard, and congruence, to develop good doctor-patient relationships [17,18]. Examples of the use of cognitive-behavioral models include the vicious circle model in the area of anxiety disorders and the influence of negative thinking and avoidant behaviors in depression. In the treatment of patients with somatoform disorders, the disease model of the patient, which has traditionally focused on physical causes, is gradually expanded to include alternative concepts of illness, and the attention is refocused on potential psychosocial stressors. In systemic thinking, interactions replace reductionist notions of cause and effect.

The teaching of doctor-patient communication skills includes learning interview techniques that are both doctor-centered (setting a time frame, introducing your own themes, interrupting, and asking closed questions) and patient-centered, allowing time for the patient to talk at the beginning of the interview, not interrupting, asking open questions, offering verbal and nonverbal encouragement to keep talking, summarizing in your own words, and reflecting emotions).

An ideal method for understanding doctor-patient interactions is the Balint group, in which the focus is placed on the transference and countertransference interactions between the doctor and patient.

### Targeted skill 4: Collaboration with mental health specialists

The fourth learning objective involves referrals to mental health specialists and cooperation with mental health services. Even in Western countries with well-developed support systems, these processes are not optimal. Primary care physicians have an important pilot function in the mental health care system. They must decide whether basic psychosomatic care is sufficient or expert assistance should be requested, as well as which expert is best suited to address the problem. The primary care physician should inform the patient of the need for more intensive psychotherapeutic and/or psychopharmacological treatment, should motivate him/her to accept such an offer and should refer him/her to the appropriate physician or facilities.

### Guidelines and didactic implementation

The guidelines for the implementation of the new curriculum:

1. There is a balanced ratio of seminars, practical exercises and patient-related self-experience.

2. Live patient interviews provide the participants with a model for conducting interviews and interventions.

3. The ability to conduct therapeutic interviews and the creation of relationships are the “red thread” running through the entire training program.

4. The basic orientation is psychodynamic and systemic. Cognitive-behavioral techniques and the basic therapeutic attitude of psychotherapy, including unconditional positive regard and respect, empathy and the congruence of the therapist as a person, will be integrated. The family-oriented point of view will be reflected in the treatment of all problems and diseases.

5. The objective is the acquisition of the individual’s psychosomatic-psychotherapeutic competence for treating the emotional and psychosomatic disorders and problems that arise in the specific area of work.

6. Theoretical knowledge, practice in interviewing and patient-related self-experience (Balint groups) will be covered by the topics in one block.

The objective of the new curriculum is the intertwining of teaching goals with learning goals and learning content. Therefore, the following teaching methods are regularly applied.

• Brief interactive lectures: This method includes practice-relevant introductions of the topics presented as PowerPoint presentations in English, with the provision of hard copies translated into the local language.

• Live patient interviews, which also include the partner or family, that provide a “real-life” experience of doctor-patient communication and diagnostic-therapeutic conversations: Patients are recruited from the daily practice of the partners. The patients’ and the families’ consent (for family and couple interviews) are obligatory for these units.

• Reflecting teams: Five to six participants come together to discuss their experiences during the live patient interviews and to provide emotional feedback to patients.

• Role-playing to teach doctor-patient communication skills: After a short impulse lecture, two teachers engage in an exemplary role-play exercise on the topic. Subsequently, the participants practice in groups of three (doctor, patient, and observer) in alternating roles. This change of perspective facilitates the consideration of a problem or disease presentation from various angles.

• Group work: Group discussions, as a didactic method of teaching, are used in small subgroups and in plenary sessions. They help to create an open climate of interdisciplinary exchange among the participants, and they provide a multi-perspective point of view of the participants’ own work. Three groups are formed prior to the live interviews, and the participants are instructed to observe the interviews with a focus on the following criteria: content, e.g., the topics that are included in the psychosocial anamnesis or the questions that are asked for the diagnostic clarification of depression; communication skills, e.g., the techniques of patient-centered and doctor-centered interviewing that are used; and doctor-patient relationships, e.g., the perceptions of the atmosphere and the level of mutual understanding, as well as the participants’ experiences, feelings, thoughts, and physical perceptions.

• Video feedback: In addition to the live experience, some well-established and helpful elements of teaching include case examples of doctor patient communication, elements of the diagnostic-therapeutic process and filmed training sessions (as feedback). Ideally, these video examples are produced with local patients and doctors. Video examples with European actors can also be translated and shown. For any examples, subtitles and written translations and explanations must be prepared.

• Sculpture: We use sculpture for a better understanding of the meaning of illness in a family system, in the doctor-patient relationship or among hospital staff. In the Asia-Link project, sculptures are used in the Balint groups to enable access to the complex interactions among the patient, the different doctors, the family members and the illness. The objective is to spatially depict a momentary situation to clarify the nearness and distance in a relationship. To achieve this goal, the reporting doctor selects persons from the group who are representative of the system, including himself/herself, and positions them in the room. Then, the teacher asks, “Who do you see? What do you feel while you’re doing this?” The representatives can be asked to describe their physical and/or emotional perceptions or to say words that spontaneously occur to them. The presenting doctor can also play the role of his own representative and experience this position for himself/herself in relation to the other participants. Afterwards, the representatives have an opportunity to find new positions that are more comfortable for them.

• Genograms: Genograms are graphic representations of a family constellation that span several generations. They show positions in the birth order, deaths, diseases, symptoms, and other life events in a concise form and are created during medical history interviews with individuals or families.

Creating a genogram involves three steps:

1. All the family members and their relationships are recorded. The process begins with the children or the couple as the core family and continues with the addition of the grandparents. In total, three generations are included, if possible.

2. In the second step, information about the family history is added: the members’ ages, sexes, marriages, divorces, miscarriages, deaths and serious diseases, and other critical family events.

3. Finally, the most relevant relationships among the family members are highlighted.

• Balint groups: In a Balint group, 8–12 doctors meet under the leadership of a trained Balint group leader. The presenting doctor describes the doctor-patient relationship. The participants listen to this report, provide their impressions and discuss their feelings and thoughts about what they have heard. This process results in a complex image of the doctor-patient relationship that the speaker can observe quietly and from a distance without speaking. He/she receives input for a new point of view, and blind spots are illuminated. He/she recognizes his/her effect on the patient and his/her own mode of behavior. Balint groups offer doctors a modicum of self-experience and teach them to pay attention to patients’ whole personalities, beyond their diseases. The result is that the patient and doctor are both more at ease. The Balint group is always the final activity of a course day.

All of the educational materials have been compiled in both digital and hard-copy tool books that are in both lecturer- and participant-friendly formats. Continuous updates and the ability to add one’s own materials enhance individual utility.

The training lasts one year and includes 60 hours of teaching. The 60 hours are divided into three blocks of 20 hours each.

During the first year, a team of prospective teachers was recruited for each center. The didactic elements of the new curriculum were taught to the future teachers. This was a mutual process that involved the German team teaching the teachers and adapting, modifying and redesigning the lessons within the context of the partners. Teaching the teachers served as an experimental curriculum while supporting the future independence of the partners from the assistance of the European experts. The experimental lessons followed the contents of the intended curriculum. Teaching the teachers, therefore, focused on three target areas:

• teaching both the content of the biopsychosocial approach in medicine and the topics included the planned curriculum;

• teaching didactic methods, strategies and skills for managing and teaching the lessons; and

• adapting the teaching methods to specific contexts.

At the end of this activity, the future teachers were able to manage the pilot curriculum with the assistance of their European partners during the second year.

During the third year, the Asian partners were encouraged to organize and implement the new curriculum on their own. The results of the evaluations and the partners’ accumulated competence helped to fit the curriculum elements to the needs of the partners. The European staff provided supervision but limited their participation in teaching the curriculum (except for roles as guest lecturers on special topics). Supervisors supported the teachers’ needs in managing the courses and becoming competent trainers of biopsychosocial skills.

More information and an overview of the 3-year program, including the experimental and supervision phases, can be found in a previously published report [19].

## Methods

At the beginning of the curriculum (T1), the participating physicians completed a questionnaire about socio-demographic data, their professional specialization and their main objectives in attending the training (see Table 1).

**Table 1:** The evaluation process

**Time**	**Population**	**Method**
T1 before training	Participants and future teachers	Questionnaire on sociodemographic data and main objectives
T2 after 1 year of training	Participants and future teachers who had received 3 blocks of training	Questionnaire concerning course evaluation
T3 after the 3rd year of training	German teachers and future teachers	Interview on the achievements of the training
T4 6 months after the 3rd year of training	Participants and future teachers	Questionnaire and open questions on the impact of the training on daily medical practice

After one year of training that included three blocks (T2), the participants, including the future teachers, rated the training using an evaluation questionnaire that asked about the relevance of the lectures and group work, the attainment of theoretical knowledge and practical skills, and respondents’ satisfaction with the organization and their own performance during the course.

To document the learning process, the German teachers and the future teachers were interviewed about the results of the training at each center at the conclusion of the third year (T3). The German teachers were asked the following questions:

1. During the third year, what were the greatest achievements and gains for the future teachers concerning theory live interviewing, reflections by the teams and observers, Balint groups, and group work? (1= no gains; 6= maximum gains.)

2. In which fields did the future teachers experience the greatest difficulties or no difficulties at all? (1= maximum difficulties; 6= no difficulties.)

3. What should be changed in advance of the next training course?

Furthermore, the future teachers were asked the following questions:

1. In what areas were your greatest achievements/successes: theory, live interviewing, the reflections of the teams and observers, Balint groups, or group work? (1= no/none; 6= maximum/best.)

2. What were your greatest difficulties in theory, skills, and didactics? (0= none; 100= maximum.)

What should be changed in terms of theory, the reflections of the teams, Balint groups, and group work in advance of or during the next training course? Half a year after the completion of the program (T4), the participants answered 8 questions about the impacts of the training program on their daily medical work and their doctor-patient communication, any difficulties they had encountered in using the skills, and any attitude changes they had experienced as doctors.

## Results and Discussion

A total of 200 medical doctors from China, Vietnam and Laos, who represented different disciplines, participated during the three years of the project (see Table 2).

**Table 2:** The training population at the beginning of the program

	**Total n= 200**	**China n= 79**	**Laos n= 41**	**Vietnam n= 80**
Female sex%	60.0	67.1	48.8	58.8
Mean age (SD)	36.7 (9.43)	35.1 (9.36)	42.2 (7.92)	35.2 (9.21)
Mean years of professional experience (SD)	11.8 (9.33)	10.6 (9.79)	15.3 (8.04)	11.3 (9.19)

The mean age of the participants was 36.7 years, the proportion of women was 60%, and they had 11.8 years of professional experience on average. Most of the participants came from the disciplines of psychiatry (n= 88), psychological medicine (n= 16) or psychosomatic medicine (n= 5). The remainder belonged to different fields of medicine, with the majority in internal medicine (gastroenterology, endocrinology, infectious disease, nephrology, or oncology) (n= 29), pediatrics (n= 15), or neurology (n= 10). Other participants practiced general medicine (n= 9), gynecology (n= 8), dermatology (n= 3), surgery (n= 2) or nursing, public health, and traditional medicine (n= 6). Thirty participants provided no information regarding their specialties.

The main objectives for participating in the curriculum are listed in Table 3.

**Table 3:** The main objectives of attending the training of 47 future teachers and/or participants (at the beginning of the first year, during the experimental phase; multiple answers and rounded percentages are presented)

**Main objectives of participating in the training**	**n**	**%**
To learn about psychosomatic medicine/biopsychosocial models	28	25%
To learn didactical teaching methods (such as interactive learning and presenting lectures)	23	20%
To acquire practical skills for daily clinical practice (such as communication, role playing, patient interviewing, family interviewing, and creating genograms)	18	16%
To learn about doctor-patient communication skills	12	11%
To improve patient management	9	8%
To learn about the diagnosis and treatment of specific psychosomatic disorders	8	6%
To address administrative and sustainability issues in the curriculum	5	4%
To learn how to use Balint group methods	5	4%
To share experiences with other doctors/acquire more self-experience	4	3%
Other	2	2%

The main objectives for participating in the curriculum included learning about psychosomatic medicine and the biopsychosocial model, learning didactic methods for teaching and improving doctor-patient communication skills for daily practice.

After one year of training that included three blocks, the participants rated the training using an evaluation questionnaire concerning the relevance of the lectures and group work, the attainment of theoretical knowledge and practical skills, and their satisfaction with the organization and their own performance during the course (see Table 4).

**Table 4:** Evaluation of the training curriculum after the first project year (the experimental phase; n= 47 future teachers and/or participants)

	**Total mean (SD)**
The psychosocial knowledge has relevance (0 being no relevance and 100 being high relevance)	
- for my daily work	66.8 (19.79)
- for my personal life	73.0 (17.80)
The skill training provided by the training courses has relevance (0 being no relevance and 100 being high relevance)	
- for my daily work	79.4 (13.43)
- for my personal life	71.3 (17.53)
The Balint groups in which I participated and the self-experience that I acquired had relevance (0 being no relevance and 100 being high relevance)	
- for my daily work	78.1 (14.54)
- for my personal life	69.3 (22.05)
Are you satisfied with your performance during the course? (from 0 being not at all to 100 being quite good)	77.8 (12.81)
Please rate the organization of the training program (from 0 being poor to 100 being quite good)	80.0 (14.60)
Please rate the didactic presentation of the training program (from 0 being poor to 100 being quite good)	81.9 (14.02)
Did the modules of the training program fit your personal needs? (from 0 indicating that the modules were too redundant or complicated to 100 indicating that my personal needs were met)	79.1 (14.12)
How important for you were the following items? (1= not important, 2= important, and 3= very important)	
- skills training	2.8 (0.40)
- theoretical knowledge	2.3 (0.59)
- self-experience	2.5 (0.59)

All of the candidates rated the training as helpful and highly relevant. The practical skills training and the Balint group work were rated particularly highly. There were no significant differences among the three countries. The cultural sensitivity issues are explained in more detail later in this article.

After the completion of the third year (the supervision phase), the future teachers and the German teachers were interviewed by G.S., who was responsible for the evaluation of the curriculum. The future teachers in Laos wanted to teach more in the future, and they wanted to translate the materials into the Lao language, taking into account transcultural issues. This group suggested that 3- to 4-day courses would be better than two days. In Vietnam, the future teachers wished for theoretical knowledge and they thought the Balint groups should be more culturally sensitive. In Shanghai, the future teachers also wanted to learn more about the theoretical background and treatment of mental disorders. They believed that future teachers should receive an official state certificate after completing the training. In Vietnam and China, the teachers desired more training, more assistance and the implementation of a psychotherapy training program. One limitation of these results is that only 1–3 future teachers were interviewed, depending on the location.

The comments of the German trainers focused on the following issues. In Laos, the participants were strongly engaged, but the preparation of the courses was unsatisfactory; e.g., there were no Lao texts. In Hue, the training sessions were well organized; in contrast, in Ho Chi Minh City, there were many logistic problems. In both cities individual group participation was not consistent. Primarily in Laos, some of the training content, such as teaching theory and live patient interviewing, was rated as less successful. In all of the other locations, the training objectives were viewed as having been achieved (see Table 5, 6).

**Table 5:** Results of interviews with German teachers (n= 3) concerning the results of the training of future teachers

**Content**	**Laos**	**Hue**	**Ho Chi Minh City**	**Shanghai**
Theory	2	5	5	5
Life interviews	2	5	5	5
Reflecting teams	4	5	5	5
Balint groups	4	5	5	5
Group work	4	5	5	5

What were the greatest achievements and gains in the third year? (1= no gains; 6= maximum gains). The data represent rounded means.

**Table 6:** Results of interviews with German teachers (n= 3) concerning the results of the training of future teachers

**Content**	**Laos**	**Hue**	**Ho Chi Minh City**	**Shanghai**
Theory	2	5	5	5
Skills	4	5	5	5
Didactics	2	6	4	5

In which fields did you perceive the greatest difficulties for the future trainers? (1= maximum difficulties; 6= no difficulties).

The main goal of the training program was to introduce medical doctors to the biopsychosocial approach and to provide them with basic skills in this approach. Two acceptable means of evaluation of training programs include 1) asking the participants about their subjective, individual, daily medical practices and 2) measuring the results directly by observing daily practices such as the time spent on doctor-patient-communication, the rate of correctly diagnosed psychosocial problems/disorders, and the effectiveness of psychosocial interventions [20-22]. The second “measurement by observation” evaluation approach was beyond the resources of this program. Therefore, we asked the participants half a year after the completion of the training program whether and how their knowledge, skills and attitudes had changed.

The results of the questionnaires and interviews can be summarized as follows (see Tables 7 and 8). In addition to practical skills for daily clinical practice, the participants wanted to learn more about didactic methods for teaching. The skills training and the Balint groups had high relevance for both the daily work and the personal lives of the participants. The organization and the didactic presentation of the training program were stimulating. The most important topics were skills training and self-experience. Half a year after the completion of the training program, the participants stated that the program had had a great impact on their daily medical work. They spent more time communicating and felt more secure about doctor-patient communication. The training program also changed their attitudes as doctors toward accepting a biopsychosocial approach to care as appropriate and doable in their medical culture and health care system.

**Table 7:** The results of the evaluation one-half year after the completion of the training program (n = 46 future teachers and/or participants; scale from 1= no to 10= yes)

	**Mean (SD)**
Did the theory/knowledge of the training program impact your daily medical work?	8.31 (1.02)
Did the skill training/Balint groups (doctor-patient communication) of the training program impact your daily work?	8.49 (0.89)
Do you spend more time on doctor-patient communication since completing the training program?	8.40 (1.21)
Do you feel competent (know how to participate) in doctor-patient communication?	8.24 (1.11)
Do you think the training program will (has) change (d) your personal practice as a doctor?	8.09 (1.79)
Are there difficulties in using the knowledge/skills of the training program?	3.43 (2.71)
1. What are your feelings concerning your daily work as a doctor? 1= burn-out to 10= I like my profession and my daily work	8.70 (1.46)
Did the training program change your attitude as a doctor?	8.07 (1.70)

**Table 8:** Written answers to the questions in table 7

In which **aspects **of your daily work, your doctor-patient communication, and your personal practice has the training program had an influence?
**Ad 1) Theory/knowledge**
Doctor-patient communication techniques
Contact with patients
Everyday clinical interviewing and educational work with students
Diagnosis and treatment
I can apply all/most the knowledge from this course to my work in dealing with difficult patients, my teaching style, and clinical interview skills
Taking histories, paying more attention to patients’ emotions when treating their problems/diseases
Teaching techniques and experience when practicing in groups, which I can apply in my work
How to include the patint’s family
**Ad 2) Skill training/Balint groups**
Empathy and sharing feelings
The Balint groups increased my understanding of patients’ problems
Contact with patients
Empathy and interpretation, hypothesizing and circularity
Obtaining information from a patient
Interviewing skills
Coping with my emotions during psychotherapy
The management of difficult cases
The patient-doctor relationship
Resolving problems
Finding new methods for handling difficulties in clinical practice
**Ad 3) Time spent on doctor-patient communication**
Anamnesis, diagnostic and therapeutic information
Diagnosis, e.g., anamnesis
I want to spend more time, but there are so many patients, and there is so little time to spend with them.
I will try to improve the doctor-patient communication in my medical practice.
All the aspects of my interviews, such as anamnesis, the gathering of information and provision of suggestions
**Ad 4) Competence in doctor-patient communication**
In dealing with suicidal, violent, or emotionally unstable patients
Forming good relationships: listening, empathy, positive feedback, and summarizing
Greeting patients, taking histories, and finding the underlying problem based on the symptoms
Yes, now I can use these doctor-patient communication skills more skillfully than ever
**Ad 5) Changes in personal medical practice**
Absolutely, it has already changed.
Greater knowledge and skill in handling psychosomatic cases
Communication with patients
It will help me improve my medical skills
Understanding more aspects of communication
Only slightly
Paying more attention to doctor-patient communication
Providing more psychotherapeutic knowledge and practical approaches
Teaching medical students
**Ad 6) Difficulties in using the knowledge/skills**
The institutional setting did not allow enough time for proper client interviews.
My experience with psychosomatic issues is still lacking.
Supervision
Lack of time
I want more teaching skills
In chronic cases (e.g. pain disorders)
**Ad 7) Feelings concerning daily work**
I can meet many people, and I can acquire experience to add to my life.
I know what I am doing and how to improve myself.
Patients distrust their doctors in China.
Receiving respect from the community
Very tired, but meaningful
**Ad 8) Changes in attitude**
About family therapy
About doctor-patient communication
How to contact patients/people
When I see the smiling faces of the patients
Greater knowledge about doctor-patient communication
More understanding of patient psychology
Not at all
Too much to express it clearly
Treating patients as clients and ways to communicate with patients and their relatives
When I face patients, I see them as whole persons.

The interviews with the future teachers and the German teachers demonstrated that, except in Laos, all of the training objectives were achieved. Despite this extremely positive overall result, future training sessions should consider the following critical points and misunderstandings and implement the following cultural adaptations.

### Misunderstandings and cultural adaptions

The results in this section are derived from the interviews with the future teachers and from debriefings of the course teachers.

#### Handling of negative emotions

Chinese culture emphasizes the inhibition of strong emotional expression [23-25]. Young children are sometimes scolded by their mothers for aggressive behavior [26]. In our communication skills training, we introduced elements of "active listening" [17,18], which include "mirroring emotions," naming perceived feelings, and showing these emotions through non-verbal displays of anger or sadness, for example. Because of their cultural background, this communication behavior was met with resistance by the participants. The essentially empathic nature of the Rogers approach was not questioned, nor was the usefulness of empathic support during difficult emotional situations, e.g., during discussions of a life-threatening disease or critical life events. However, the expression of personal concerns or feelings in difficult doctor-patient relationships was rejected, e.g., if the patient were upset and angry because he/she experienced a prolonged wait. In this case, a statement such as "I can understand that you are very angry" or "If I were you, I would be upset, too" would be considered an admission of guilt by the doctor. Such an admission would be associated with a loss of authority over the patient and could cause the patient to change doctors.

#### Balint groups, sculpture work, and reflecting teams

In the Asian countries, it is not customary to speak directly about personal feelings when discussing a case in a Balint group. It is also foreign to some Asian cultures to discuss negative feelings outside the family [25]. During the first Balint group sessions, these traditions were repeatedly confirmed by the future trainers. Our experience with classical Balint work appeared to confirm that the participants felt overwhelmed by the instruction to express their thoughts, feelings and fantasies freely. Subsequently, we modified the classical Balint work by the introduction of sculpture. This modification completely changed the results: the participants became absorbed in their roles; they spoke of their fears, their anger, and their sadness; and they identified with the people they represented. In this way, sculpture facilitated vivacity and improved doctor-patient dynamics. Hidden conflicts became emotionally palpable, and ideas for possible solutions were clarified. The doctor-patient relationship was part of the system and was improved by a change in the position of the doctor, which resulted in decreased symptoms and improved well-being on the part of the patients.

Similar to the sculpture work, the systemic techniques, such as the reflecting teams and the genograms, were perceived as enriching in all three countries. The genograms were recorded on flipcharts during the live patient interviews, initially by a German teacher and later by a future teacher, and they were subsequently included in the case discussions. The participants in the reflecting teams were selected prior to the live patient interviews. Following the interview, but in the presence of the patients, these participants provided empathic and supportive feedback. During the exchanges of the reflecting teams, in particular, the patients felt deeply moved and expressed their gratitude for the emotional understanding that they had received. In addition, during the reflecting teams, the processing of grief, anger and insults sparked memories of personal experiences that added to the responses. This process led to brief moments of consolidation of the patients’ and participants’ experiences, which could not be achieved solely by a plenary discussion of the case.

#### Family norms and "breaking bad news"

For the physicians in the three countries involved in the program, disclosing diagnoses and prognoses to patients is extremely challenging. Most Chinese families ask doctors not to reveal the diagnoses and prognoses of family members [27]. One reason for this request might be families’ fear that a loved one suffering from cancer will despair and become hopeless upon learning of their diagnoses [28]. The methods of delivering bad news in oncology are influenced by cultural diversity [29]. In Western countries, oncologists usually inform cancer patients of their diagnoses [30]. In addition, 98% of patients in Western countries want to know their diagnoses, and 87% of patients wish to be made aware of all possible information, both good and bad [31].

When patients follow the principle of autonomy and families follow the principle of beneficence in disclosing information about cancer, physicians find themselves in a dilemma [32]. Chinese doctors need support in solving this dilemma and determining what to tell to cancer patients. In our communication skills training, we addressed this dilemma and tried to find solutions for it in specific cases. We must find a balance between disclosing information about cancer and the needs of patients and families. We took seriously physicians’ concerns about disclosing information about cancer diagnoses, and we carefully sought ways to solve the conflict between the patient’s right to be informed and the traditional norms of the Asian family without harming the patient. Western concepts of breaking bad news are rooted in patient autonomy and are not easily transferable to Asian cultures. In Asian societies, the family, not the individual, is the basic structural and functional unit of society; affiliation with a social group, such as the family, has greater value than in Western societies. In Asian cultures, the principles of family decision-making and family involvement in decision-making are paramount and constitute an integral part of the self-concept of the individual [33]. This cultural characteristic should be appropriately recognized and acknowledged.

The dilemma of patients’ autonomy and traditional norms has not yet been resolved. The traditional means of handling this dilemma no longer apply, which is also due to the increasing demands of patients to be better informed. The way this dilemma is handled in Western countries is not easily transferable to Asian societies, mainly because of the importance of the family in the latter. Future training sessions will continue to address this issue, in an attempt to find a family-centered approach [34] of breaking bad news to the patient and his/her family.

#### Doctor-patient relationship

The doctor-patient relationship in all three Asian countries can be described as patriarchal and is characterized by a doctor-centered mode of communication. Patients expect the doctor to be the expert and to tell them what to do. Patient-centered interviewing is unusual in Asia.

The patients in the live patient interviews and the participating physicians appreciated the empathic, respectful, patient-centered interview style. However, the German teachers were also considered "famous professors from abroad," from whom cures for their ailments were expected. Due to the traditional norms, a cooperative doctor-patient relationship with a patient-centered interview style might not be immediately implemented in everyday life. Elements of patient-centered communication skills such as short breaks, summarizing, and questions regarding subjective health have proven to be highly teachable and learnable as a first entry into different attitudes and a better design of the doctor-patient relationship.

#### High performance pressure

In most general outpatient clinics and in outpatient psychiatric and psychosomatic medicine clinics, 30–60 patients are seen by one doctor in a single morning; in large hospitals in China, a total of up to 10,000 patients per day are seen. Usually, the doctor has only 2–3 minutes for each patient. In specialty outpatient clinics, which are usually operated by experienced specialists and are rather expensive, the doctors might have 10–15 minutes. This high performance pressure raises doubts about whether the diagnostic and therapeutic skills learned in the training sessions can be applied in everyday practice. We recommended that the participants initially gather psychosocial histories from selected patients and provide supportive care only when there is a sufficient time frame of 10–15 minutes.

#### Language

One of the conditions for selection as a "future teacher" included good English-language skills. This condition could not be satisfied in any of the three countries. Particularly in Laos, most doctors only had a rudimentary knowledge of English. In all three countries, the participants constantly relied on the translations of English-speaking participants. It was difficult for us to assess the quality of these translations. Based on feedback from the participants, the quality ranged from excellent to extremely poor. Especially during the doctor-patient interviews, there were frequent protests by the participants when certain passages were only partially translated from the local language to English. Here, censorship may have occasionally occurred without our notice. It is important to make all of the materials available in English and in the respective local languages so that all of the participants can follow the course smoothly. In this way misconceptions, such as in the translation, can be clarified. Many participants with insufficient knowledge of English feel ashamed to ask questions.

#### Disease patterns

Our selection of mental disorders and problem areas coincided with WHO data on the most common mental disorders [1,6,8]. In particular, patients who have physical complaints without adequate organ findings (somatoform disorders) are common in all three countries. Because psychosocial conflicts are rarely expressed directly, emotional distress is expressed through physical symptoms. In Laos, there was a desire to include cancer, high blood pressure and stroke as physical illnesses associated with respective psychosocial problems. The 2008 earthquake in China led to a greater demand for knowledge about and intervention in post-traumatic stress disorder (PTSD). Following the Vietnam War and the Cultural Revolution in China, PTSD could be considered a disease pattern in all three countries; however, scientific data on this issue are not available. Our partners did not seek help addressing PTSD resulting from war and persecution.

#### Chaos and disorganization vs. German thoroughness

The lack of continuity on the part of the participants caused problems for the local organizers. Often, doctors were absent in the afternoon without prior consultation or excuse. New participants joined the course during the second of the 3 blocks; however, they had no familiarity with the first part of the course. During the second and third years, however, this discontinuity was mitigated somewhat by prior participation. Therefore, at least at the level of the future teachers, there was continuity. The reasons for the sudden disappearance of some of the participants were not, as we had initially suspected, due to disinterest but rather the arbitrariness of the hierarchical system. The sudden organization of meetings, the delegation of tasks that must be accomplished quickly, and last-minute travel to other cities were common in all three countries. Flat hierarchies and the autonomy of the individual employee in these countries, as we know them in Western countries, were rare. Furthermore, noncompliance with agreements and sudden changes in agreed-upon time frames can lead to confusion. We learned that long-term agreements did not have the same binding force as in Western countries. Decisions were made for the present moment and could change by the following day. This difference required great flexibility on the part of the German teachers. However, the relinquishment of preconceived concepts and schedules was also associated with more freedom and ease and made it possible to simply “go with the flow.”

#### Impacts on the health care system

Primary care is part of community hospital health center-based services [14]. In outpatient clinics, there is little room for individual, personal doctor-patient relationships, which frequently form the basis of successful treatment. Somatizing patients in China often complain about a lack of understanding of their complaints by their doctors and about a lack of treatment success [35]. Conversely, a WHO study of mental disorders in primary care showed that patients with continuous doctor-patient relationships presented with somatic symptoms as frequently as those who did not experience such relationships [6,36,37]. Continuous doctor-patient contact and good doctor-patient relationships should be implemented, comparable to the role of the general practitioner in primary care services in Europe and other Anglo-American areas.

There were only a few participants who were trained in traditional medicine. These participants were very interested in the biopsychosocial approach and felt that the integration of psychosocial interventions into their work was helpful. However, this level of interest was not representative of participants in general clinical practice, where there is little cooperation between traditional medicine and mental health services. Nonetheless, the relationship between Western medicine and traditional medicine continues to develop. The holistic approach of psychosomatic medicine meshes with the universal approach of traditional medicine. However, individual psychodynamic thinking is not part of traditional medicine; rather, emotions are assigned to specific organs. Mental disorders, like physical disorders, are considered to be the result of disturbances in energy flow. In addition, the doctor-patient relationship is designed hierarchically in traditional medicine. Little attention is paid to traditional illness attributions or to notions of the relationship between patients and doctors. This discrepancy might be partially due to our classes being held in Western-oriented cities, although patients in Vietnam and Laos frequently travel for several hours from the countryside to the city.

In future courses, a more detailed medical history of subjective illness conceptions and expectations would certainly expand our range of knowledge. Future training programs should also include rural areas. In the future, these cultural blind spots can be better addressed through our new insight about these potential misunderstandings and close cooperation with our partners [38-43].

In Shanghai, the training course provided state-approved continuing medical education credits (CMEs), which enabled the Department of Psychosomatic Medicine to view it as an attractive instrument for the establishment of psychosomatic medicine as a regular postgraduate training topic for medical doctors. In Ho Chi Min City and Hue, the curriculum is now a valid unit for the point-credited postgraduate medical training system for faculty. Regarding the mental health sector, the situation in Laos is fundamentally different from that in China and Vietnam. Due to fewer resources, the prospects for the development of psychosomatic medicine in Laos were slight. The mental health services in that country were poorly developed, and there were few doctors who might qualify as future teachers. Interest in the program was primarily exhibited by general practitioners; however, there was little continuity in attending the training classes. In Laos, it was not possible to continue the training program after the completion of the project phase due to a lack of commitment by the partners. Our experiences in Laos demonstrate that future training programs should also incorporate psychiatric disorders, such as schizophrenia, organic brain disorders and addictions.

#### Current and future projects

In China and Vietnam, advanced training in psychosomatic medicine and psychotherapy has begun. Tongji University in Shanghai, together with the medical faculty of Freiburg, Germany, launched a Master’s degree in Psychosomatic Medicine and Psychotherapy in September 2011. In Vietnam, a 4-year-program began in November 2011 with three objectives: (i) the introduction and implementation of a 3-level, modular curriculum on psychosomatic medicine and psychotherapy for medical students and doctors; (ii) the establishment of this curriculum as a regular part of the national curriculum of medical education on psychosomatic medicine and psychotherapy; and (iii) the development of international European-Asian networks and the enhancement of intercultural communication.

The ASIA-LINK curriculum has also promoted the development of joint research projects and the exchange of doctors and medical students between Germany and China and Vietnam. Through contact with the Cancer Hospital in Beijing, a training program for Chinese oncologists, "Breaking Bad News," has been developed and scientifically evaluated [44]. The German Robert Bosch Foundation, the German Research Council and the Sino-German Center for Research Promotion in Beijing have supported research projects on illness perception, illness behavior, doctor-patient-relationships and the treatment outcomes of patients with medically unexplained physical symptoms (somatoform disorders) in China.

Finally, the experiences with Balint groups in our program led to the establishment of a Chinese Balint society. The first conference, which included international participation, occurred in May 2011. The participants expressed a desire to establish Balint groups at their own hospitals, which has led to a plan to offer a Balint group leader seminar at the next international conference, in June 2012.

## Conclusions

This project had a significant impact on all of the participants. The course evaluation demonstrated that the medical doctors enrolled in the courses received highly qualified and effective training and demonstrated the participants’ progress in both professional competence and self-development. These physicians are now connected by international collaboration and intercultural cooperation. The establishment of national and international networks has been facilitated. The future teachers learned practical clinical skills and didactic and management skills in applying the training curriculum. They have taken over key responsibilities in continuing and consolidating the future training and education of medical doctors in their countries. The partner university hospitals, as institutional stakeholders, have shown interest in the project and have granted support for further development in this field. The European partners gained valuable experience and competence in international projects and in the successful conduct of intercultural communication. As a result of the exchanges of ideas and practical experience between the Western and Asian medical doctors and patients, new elements can be added to Western treatment theories in Germany, China, Vietnam and Laos that will help to create promising new research fields. Finally, patients will benefit from an improved mental health care system.

## Competing interests

The authors declare that they have no competing interests.

## Authors’ contribution

Kurt Fritzsche carried out the training, participated in the evaluation and drafted the manuscript. Peter Scheib was a protect leader, he was responsible for the organisation and the training. Nayeong Ko has made substantial contribution to conception and design. Michael Wirsching has made substantial contribution to conception, design, analysis and interpretation of data. Andrea Kuhnert made substantial contribution to conception and design, and was involved as a trainer. Jie Hick participated in the design of the study and performed the statistical analysis. Gerhard Schüßler was responsible for the evaluation and performed the statistical analysis. Wenyuan Wu had made substantial contribution to conception and design. Shen Yuan has made substantial contribution to conception and design. Nguyen Huu Cat has been involved in drafting the manuscript and revising it critical for important intellectual contents. Sisouk Vongphrachanh participated in its design and coordination and helped to draft the manuscript. Ngo Tich Linh participated in its design and coordination and helped to draft the manuscript. Ngyuen Kim Viet participated in its design and coordination and helped to draft the manuscript. All authors read and approved the final manuscript.

## Authors’ information

ASIA-LINK workgroup

Yuan Shen, (Shanghai), Chumbo Li (Shanghai), Duc Dang An, (Ho Chi Minh City), Ngoc Tich Linh (Ho Chi Minh City), Ho Dzung, (Hue), Cao Ngoc Thanh (Hue), Sing Menorath (Vientiane), Bettina Engemann and Annette Paskert (Germany).
